# Cost-effectiveness of voretigene neparvovec in the treatment of patients with inherited retinal disease with RPE65 mutation in Switzerland

**DOI:** 10.1186/s12913-022-08211-y

**Published:** 2022-06-28

**Authors:** Arjun Bhadhuri, Daniel Dröschel, Mike Guldimann, Claudia Jetschgo, Judit Banhazi, Matthias Schwenkglenks, C. Simone Sutherland

**Affiliations:** 1 Institute of Pharmaceutical Medicine (ECPM), University of Basel, Klingelbergstrasse 61, CH-4056 Basel, Switzerland; 2 Novartis Pharma Schweiz AG, Rotkreuz, ZG Switzerland; 3 Novartis Pharma AG, Basel, Switzerland

**Keywords:** RPE65, Cost effectiveness, Health Economics, Inherited retinal disease, Voretigene neparvovec, Switzerland

## Abstract

**Objective:**

We aimed to evaluate the cost-effectiveness of voretigene neparvovec (VN) compared with standard of care (SoC) for patients with inherited retinal disease (IRD) caused by a biallelic *RPE65*-mutation. VN is a live, non-replicating adeno-associated virus serotype 2 (AAV2). SoC is best supportive care provided to patients with visual impairment. Patients under SoC may experience progressive vision loss leading to complete blindness.

**Methods:**

We adapted a previously published Markov cohort model for IRD. An annual cycle length, life-long time horizon, discount rate of 3% for cost and health outcomes, and Swiss health system perspective were used. Data from a randomised controlled phase III trial of VN versus SoC (ClinicalTrials.gov: NCT00999609) were used to estimate transitions between health states in the first year, after which VN patients were assumed to remain for 39 subsequent years in the health state they were in at the end of the first year. After the 40^th^ year for VN patients and 1^st^ year for SoC patients, visual decline was modelled based on observational data on the natural progression of the disease. Quality-adjusted life years (QALYs) were calculated based on an external study which elicited clinicians’ EQ-5D-5L-based utility estimates for IRD patients with a *RPE65*-mutation. Costs (Swiss Francs (CHF), year 2018-2019) included drug acquisition/ administration, adverse events, testing for sufficient viable retinal cells, and healthcare-related costs of blindness. Societal costs of blindness were added in a complementary analysis. Robustness of the model results were tested in sensitivity and scenario analyses.

**Results:**

For the base-case, VN resulted in incremental costs per patient of CHF 764’402 (VN: CHF 901’654, SoC: CHF 137’252), incremental blindness-free years of 7.67 (VN: 28.32, SoC: 20.65) and incremental QALYs of 6.73 (VN: 18.35, SoC: 11.62), leading to an incremental cost-effectiveness ratio of CHF 113’526 per QALY gained. In probabilistic sensitivity analysis, the cost-effectiveness of VN was better than CHF 100,000 per QALY gained in 41% of iterations. For the scenario analysis in which a societal perspective was adopted and for which a 50% work-related productivity loss from blindness was assumed, incremental costs of CHF 423,837 and an ICER of CHF 62’947 per QALY gained were produced. The scenario assuming VN treatment effect lasts for 20 years produced an ICER of CHF 156’171 per QALY gained, whereas assuming a life-long VN treatment effect resulted in an ICER of CHF 96’384 per QALY gained.

**Conclusion:**

The incremental cost-effectiveness ratio of VN compared to the SoC was estimated to be CHF 113’526 and CHF 62’947 per QALY gained, respectively, from a Swiss healthcare system, and societal perspective assuming a 50% productivity loss.

**Supplementary Information:**

The online version contains supplementary material available at 10.1186/s12913-022-08211-y.

## Introduction

The term inherited retinal dystrophies (IRD) relates to a group of diseases caused by genetic defects that lead to blindness [1]. Retinitis pigmentosa (RP) is the most common IRD [2], while Leber congenital amaurosis (LCA) is another type of IRD which is characterised as one of the most severe IRD forms [1]. The occurrence of mutations in the retinal pigment epithelium-specific 65 kDa protein (RPE65) gene (henceforward, RPE65 mutations) within retinal dystrophies is rare by Swiss standards [3], and these mutations may cause RP or LCA. Before VN, there was so far no treatment available for patients diagnosed with RP or LCA caused by RPE65 mutations.

IRDs impose significant costs, particularly in relation to wellbeing costs, productivity costs and informal carer costs. In a recent study, total costs attributable to IRDs were estimated at GBP 523.3 million per year in the United Kingdom, with this estimate comprising both economic costs (GBP 327.2 million per year) and wellbeing costs (GBP 196.1 million per year) [4]. Individuals with RPE65-mediated IRDs are visually impaired at low levels of lighting from infancy and the majority become fully blind in adulthood [5].

Voretigene neparvovec (VN, Luxturna®), a gene therapy using an adeno-associated viral vector, aims to restore or maintain patients’ vision and delay progression by inserting a functioning copy of RPE65 into the cell [6]. VN is a one-time subretinal injection per each eye. It has demonstrated long-term improvements in visual function (i.e. visual acuity, visual field, full-field light sensitivity threshold) and functional vision (the ability to navigate in daily life) [7, 8]. This was shown in an open-label, randomised, controlled phase 3 trial of 31 patients performed at two sites in the USA, with an intervention arm in which 20 patients received bilateral VN treatment and 1 patient withdrew after randomisation, and a control arm consisting of 9 patients who participated and 1 patient who withdrew after randomisation (Study 301) [7–9]. After one year, the 9 participating patients in the control group were eligible to receive VN treatment (Study 302) [10]. The treatment regimen of VN for one eye begins with a course of oral prednisone, followed by an outpatient subretinal VN injection, followed by another course of oral prednisone; the process is then repeated for the other eye.

Because patients with biallelic RPE65-mediated IRD and with sufficient viable cells for VN treatment is rare (there are only approximately 13 patients in Switzerland currently eligible for VN treatment [11–16]), the absolute costs and benefits to society from VN treatment may be considered to be small, even though VN treatment is important to the individual. However, high-cost treatments are currently becoming available for various ultra-orphan diseases. Therefore, the overall economic impact of these treatments may be of interest to decision makers in the health care system. Comparisons with other treatments for an ultra-rare disease are rarely feasible. Additionally, cost-effectiveness analyses constitute an appropriate approach to put high one-time costs in relation to expected long-term benefits. If incremental cost-effectiveness ratios (ICERs) are too high for a certain treatment, money may be better put to use for treatments in another area of the health care system. By 2024, orphan drugs are forecast to represent 20.3% of worldwide prescription sales, and orphan drug sales are expected to grow at approximately double the rate foreseen for the non-orphan drugs market [17].

VN has been licensed by Swissmedic [18], by European Medicines Agency [19] and the US Food and Drug Administration [20]. Further, the National Institute for Health and Care Excellence (NICE) recommended VN for use in the National Health Service in England and Wales [21]. Its cost-effectiveness has not yet been assessed for Switzerland.

## Objective

The objective of this study was to assess the cost-effectiveness of VN in the Swiss setting, from a healthcare system and a societal perspective. As IRD is considered a birth defect in Switzerland, the initial decision on the reimbursement of VN for patients below 20 years is expected to be taken by the Swiss Federal Social Insurance Office, not by the Swiss Federal Office of Public Health, which is responsible for the compulsory health insurance system.

## Methods

The cost-effectiveness analysis for Switzerland was carried out by adapting (i.e. validating and re-parametrising) a cost-effectiveness model previously developed and implemented for the UK [22], to inform a Highly Specialised Technology Appraisal by NICE [23]. VN treatment was compared against the current standard of care (SoC). SoC involves patients not receiving VN treatment and instead receiving best supportive care (such as visual aids, technical aids, support and care in daily life). The primary health economic outcome was the incremental cost-effectiveness ratio (ICER), expressed as cost per quality-adjusted life year (QALY) gained. Additional outcomes were life years, blindness-free life years, and total and disaggregated costs. After the first year, costs and QALYs were discounted by 3% per year. The time horizon was 85 years to represent the entire lifetime of patients, and the cycle length was one year.

### Model structure and population

**Fig. 1:**
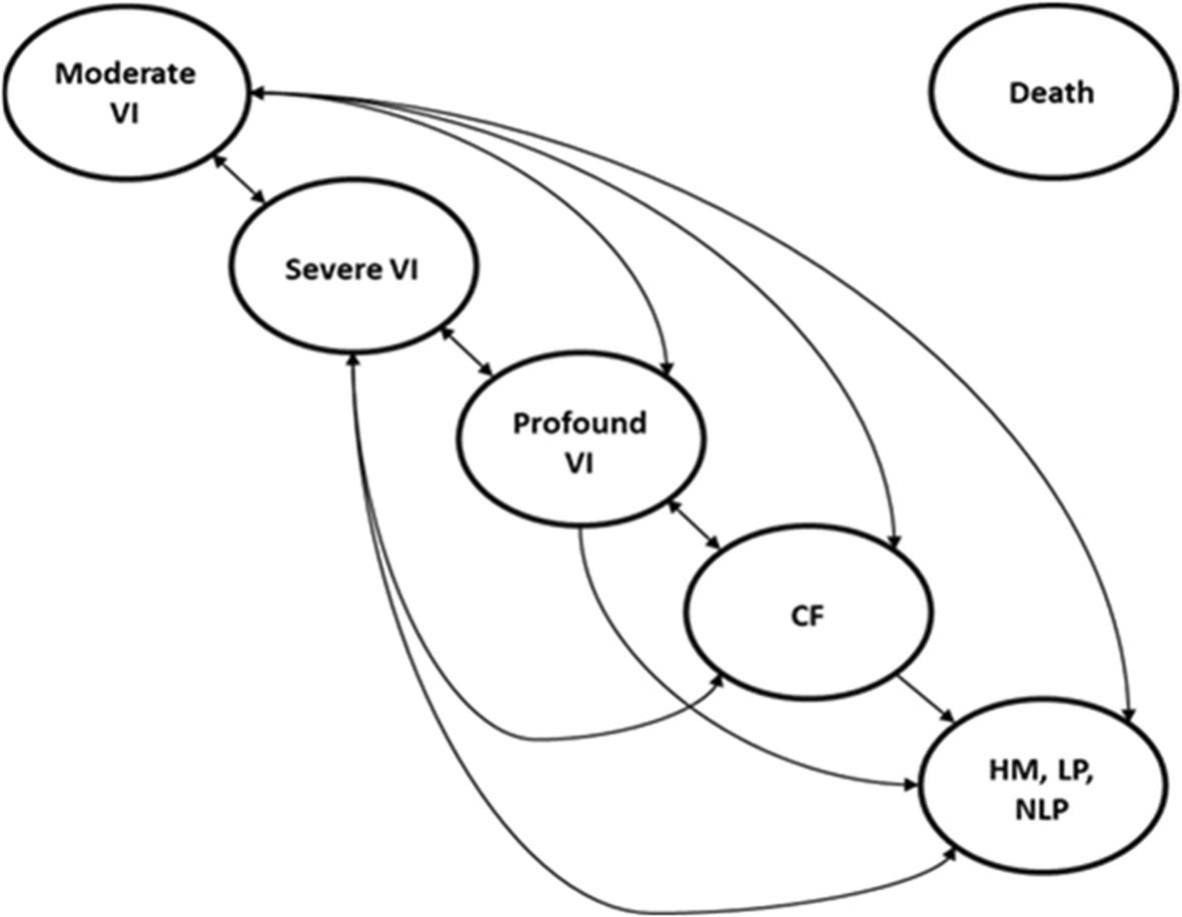
Markov model structure, taken from National Institute of Health and Care Excellence report [23]. KEY: CF, count fingers; HM, hand motion; LP, light perception; NLP, no light perception; VA, visual acuity; VF, visual field; VI, visual impairment

The model’s Markov structure considers five health states (HS) related to visual acuity (VA)/ visual field (VF), and a death state. The best vision-related health state patients could be in was 'moderate visual impairment' (i.e. VA>1 on LogMAR (Logarithm of the Minimum Angle of Resolution) scale as the average of both eyes and/or VF>240 degrees as the average of both eyes) and the worst was 'hand motion, light perception or no light perception' (i.e. VA≥3 on LogMAR scale as the average of both eyes, or indications of hand motion, light perception or no light perception across both eyes). Criteria used for categorising patients into a health state were derived from American Medical Association guidelines and are provided in appendix Table 1. Transitions between health states are presented in Fig. 1. During each model cycle, patients were simulated to either remain in the same health state, transition to a better (only possible in the first year) or worse vision-related health state, or die.

The population of interest were IRD patients with a biallelic *RPE65*-mutation and sufficient viable retinal cells. Patient characteristics were assumed to reflect those of the Study 301/302 (see below) trial population [8], with a mean age of 15.1 years at baseline, 23% of patients in HS1, 32% in HS2, 23% in HS3, 19% in HS4 and 3% in HS5.

### Clinical effectiveness parameters

The clinical effectiveness of VN treatment was based on Study 301/302 trial data (ClinicalTrials.gov: NCT00999609) [7, 8, 10]. Study 301/302 is the only Phase 3 randomised controlled trial of VN for IRD patients with a biallelic *RPE65*-mutation. The study found a statistically significant improvement in multi-luminance mobility testing (MLMT) performance (primary outcome) at 1-year follow-up (MLMT improvement=1·6; 95% CI: 0·72 to 2·41). The MLMT measures functional vision, by assessing the ability of an individual to navigate an obstacle course accurately and at a reasonable pace at different levels of illumination.

The Markov model consisted of two phases to encompass the lifetime horizon of the analysis: an initial phase for the first year, and a long-term phase for the subsequent 84 years. Transition probabilities for the initial phase were calculated by assessing VA/VF outcomes in the first year of the Study 301/302 trial, with a further assumption made for patients in HS5 at baseline (Table 1). This assumption was made because no Study 301/302 patients in the VN or SoC strategies who were in HS5 at baseline were observed at 12 months (due to participant withdrawal). Therefore, it was assumed that for the initial phase, transition probabilities for HS5 patients at baseline would move in the same direction and same number of steps as the HS4 patients in each treatment strategy (e.g. as 100% of SoC patients in HS4 at baseline were in HS3 at 12 months, it was assumed 100% of SoC patients in HS5 at baseline were in HS4 at 12 months).

**Table 1:** Transition probabilities for initial phase of model (between baseline and 12 months)

		STANDARD OF CARE STRATEGY				
		Health state at 1-year				
		**HS1**	**HS2**	**HS3**	**HS4**	**HS5**
Health state at baseline	HS1	1.00	0.00	0.00	0.00	0.00
	HS2	0.25	0.50	0.00	0.25	0.00
	HS3	0.00	0.00	1.00	0.00	0.00
	HS4	0.00	0.00	1.00	0.00	0.00
	HS5	0.00	0.00	0.00	1.00	0.00
		VN STRATEGY				
		Health state at 1-year				
		**HS1**	**HS2**	**HS3**	**HS4**	**HS5**
Health state at baseline	HS1	1.00	0.00	0.00	0.00	0.00
	HS2	0.83	0.17	0.00	0.00	0.00
	HS3	0.50	0.50	0.00	0.00	0.00
	HS4	0.50	0.00	0.25	0.25	0.00
	HS5	0.00	0.50	0.00	0.25	0.25

All visual acuity scores measured by logMAR (VA) and visual function (VF) scores measured by sum total degrees, are calculated as the average of both eyes.

HS1, moderate visual impairment (patient either has VA<1 or VF>240; and does not belong to a worse health state); HS2, severe visual impairment (patient either has VA≥1 and VA<1.4, or VF≤240 and VF>144; and does not belong to a worse health state); HS3, profound visual impairment (patient either has VA≥1.4 and VA<1.8, or VF≤144 and VF>48; and does not belong to a worse health state); HS4, counting fingers (patient either has VA≥1.8 and VA<3, or VF≤48; and does not belong to a worse health state); HS5, hand motion, light perception to no light perception (patient has VA≥3, or indications of hand motion, “light perception”, or “no light perception” across both eyes)

*BSC* best supportive care, *TP* transition probability, *VN* voretigene neparvovec

After year 1, for VN patients the VN treatment effect was assumed to be retained for the next 39 years. This meant that apart from the VN patients projected to transition to death, for 39 years VN patients were assumed to remain in the same health state that they were in at the end of year 1. A treatment effect duration of 40 years was assumed as it represents the midpoint between a minimum of 7.5 years (based on available Phase 1 trial follow-up data for VN [24]), and a maximum of 70 years (assuming the treatment effect lasts for the entire lifetime of patients [23]). 40 years was accepted to be the most plausible treatment effect duration by the NICE committee for the UK VN submission [23]. Transition probabilities representing visual decline during the long-term phase were based on a Weibull survival regression derived from a retrospective dataset of 70 IRD patients with *RPE65*-mutation (Appendix Table 2) [25, 26]. The Weibull model was chosen as it exhibited best statistical fit (assessed by Akaike and Bayesian information criterions) out of the different distributions tested. The Weibull regression was applied after the 1st year of the model for SoC patients and applied after the 40th year for VN patients. The multistate Weibull regression model used provides estimates of the transition intensities (which are converted to transition probabilities) at each cycle in the economic model. This effectively means that in each cycle a different transition probability matrix is used. The transition probabilities are therefore time-dependent, however this ‘time’ is time from baseline (and not time since entering a health state). The assumption made is the classical Markovian assumption of memorylessness and therefore this does not require knowledge of time spent in each health states. All transition data was pooled to inform a single multistate survival model which allowed the calculation of transition probabilities in each cycle based on time from the start of the model. An overview of the use of multi-state models for the analysis of time-to-event data is provided by Meira-Machado et al [27]. A reference transition from HS1 to HS2 was used, and hazard ratios representing other transitions (e.g. from HS1 to HS3) applied to calculate the probability of making each transition. Mortality was modelled based on Swiss lifetables [28].

### Utility inputs

Health state utilities were derived from a study where EQ-5D-5L estimates for IRD patients with biallelic *RPE65*-mutation were elicited from clinicians (it was not considered feasible to collect EQ-5D-5L data directly from a representative sample of patients due to the rare nature of the disease) (Table 2) [5]. Utility decrements resulting from the adverse events in Study 301/302 were included for the adverse events which occurred in at least 2 patients, could be attributed to VN treatment/administration procedure, and are associated with a utility reduction [22]. These adverse events were cataracts, eye inflammation, and increased intraocular pressure (IOP), and were incorporated into the analysis in the form of a one-off QALY loss for VN patients (Table 3). The proportion of VN patients affected by these adverse events were taken from Study 301/302. The utility decrements of cataracts and eye inflammation and assumed duration of these adverse events, were obtained from a NICE report estimating these decrements for age-related macular degeneration patients [29]. Due to the absence of a study estimating the utility decrement of increased IOP, it was assumed to be the same as the utility decrement of uncontrolled/severe glaucoma which was obtained from a study by Pershing et al [30]. Duration of increased IOP was assumed to be one month as all increased IOP events in study 301/302 were fully resolved by 1 month.

**Table 2:** Health state utility values

Source	HS1	HS2	HS3	HS4	HS5
Acaster Lloyd (EQ-5D-5L) [5]	0.71	0.62	0.52	0.35	0.15
Acaster Lloyd (HUI3; used in scenario analysis) [5]	0.52	0.36	0.22	0.14	-0.04

Key: HS1, moderate visual impairment; HS2, severe visual impairment; HS3, profound visual impairment; HS4, counting fingers; HS5, vision ranging from being able to see hand motion to having no light perception at all.

*EQ-5D-5L* five-level version of the EQ-5D-5L instrument, *HS* health state, *HUI-3* Health Utilities Index Mark 3.

**Table 3:** Adverse event disutilities

Event in VN arm	Utility decrement	Duration (months)	Proportion of patients
Cataract	0.14	1.0	15%
Eye inflammation	0.30	3.6	10%
Increased IOP	0.10^**a**^	1.0	20%

^a^ Assumption, as no disutility data associated with increased IOP were identified.

*AE* adverse event, *IOP* increased intraocular pressure.

### Costs

In the base-case analysis, for the VN strategy health-care costs included eligibility testing, VN acquisition, administration of VN and treatment of adverse. For both treatment strategies, costs included those generated due to impaired vision, in the form of technical assistance including vision aids, community and residential care services for visually impaired patients over 65 years of age. All resource use parameters were taken from published studies, and – if required – verified to be applicable to Switzerland by Swiss clinical experts (Table 4). Unit costs for outpatient and inpatient services in Switzerland were derived from national sources [28, 31–37] (Table 4). The planned public price of VN (Swiss francs (CHF) 759'968) was used in the analysis. In a complementary analysis, a societal perspective was adopted. In this, indirect costs in the form of productivity losses from impaired vision, in the workplace for patients and caregivers, as well as increased education costs for patients aged below 18 years were additionally included. These were estimated from Swiss national statistical data [31, 35, 36].

**Table 4:** Unit costs and resource use values

Cost element	Resource use: number and unit description (where relevant)	Unit cost (CHF)	Sources
VN treatment			
Acquisition cost (public price) of VN	1	759'968	Novartis
Cost of retinal surgery	2 administrations (1 per eye)	3474	[25]; unit cost Swiss-specific: [38]
Cost of prednisone	Regimen cost for both eyes	86	[25]; unit cost Swiss-specific: [32]
Cost of control visits with optical coherence tomography	4	271	[25]; unit cost Swiss-specific: [38]
Adverse events			
Cataract	Once in 15% of VN patients	4’869	[7, 8, 10, 25]; unit costs Swiss-specific: [37, 38]
Eye inflammation	Once in 10% of VN patients	52	[7, 8, 10, 25]; unit costs Swiss-specific: [38]
Elevated intraocular pressure	Once in 20% of VN patients	220	[7, 8, 10, 25]; unit costs Swiss-specific: [38]
Testing prior to VN treatment			
Testing for viable retinal cells	All IRD patients with RPE65 mutation are tested. It is estimated by medical examiners in Study 301/302, that 55% have sufficient viable cells and 45% do not	271 / 0.55 = 493	[25]; unit costs Swiss-specific: [38]
Costs of visual impairment			
Excess hospitalisations in HS2-5 patients aged 65+ years	Compared to HS1 patients aged 65+ years, 0.2 additional hospitalisations per year	12’543 per hospitalisation	[39] Unit costs Swiss-specific: [34, 35]
Technical assistance (including vision aids)	Relative levels of resource use estimated at 1.00 for all HS1 patients, 0.96 for HS2-5 patients aged 18-64 years, and 1.34 for HS2-5 patients aged 65+ years	2’133 per year	[40] Unit costs Swiss-specific: [33]
Community care (Spitex)	6% of HS2-5 patients aged 65+ years	7’063 per year	[41] Unit costs Swiss-specific: [36, 42, 43]
Residential care	30% of HS2-5 patients aged 65+ years	64’537 per year	[41] Unit costs Swiss-specific: [36, 42, 43]
Societal costs (scenario analysis)			
Excess education costs of HS2-5 patients aged below 18 years	All relevant patients	21’094 per year	[44] Unit costs Swiss-specific: [31, 35]
Productivity loss of HS2-5 patients aged 18-64 years	80% reduction to average worker’s annual salary of CHF 42,843	42’843 per year	[45] Unit cost Swiss-specific: [35, 36]
Productivity loss of family caregivers of patients aged 65+ years	Annually, 144 hours for HS1 patients, 676 hours for HS2 patients, 1608 hours for HS3-5 patients; conservatively accounted for only 25% of caregiving hours for all patients.	43 per hour	[46] Unit cost Swiss-specific: [35]

*KEY*: *AE* adverse event, *CHF* Swiss francs, *HS* health state, *N/A* not applicable, *PSA* probabilistic sensitivity analysis, *RU* resource use, *VN* voretigene neparvovec

In order to assess the robustness of the results, a univariate sensitivity analysis and a probabilistic sensitivity analysis (PSA) of the base case results for the healthcare system perspective were performed in addition to a range of scenario analyses.

### Sensitivity and scenario analyses

In the univariate sensitivity analyses, parameters were varied by their 95% confidence limits if available, and otherwise by ±20%. The following groups of parameters were included: Weibull regression parameters representing visual decline in the long-term phase, parameters representing health state utilities or adverse event related utility decrements, parameters representing duration and probability of AEs, resource use parameters, and cost parameters (excluding the acquisition cost of VN, which was treated as fixed). A Tornado diagram was produced; the presentation of results were restricted to the 10 most influential parameters.

A probabilistic sensitivity analysis using 10’000 iterations was performed. Normal distributions were used for the Weibull regression parameters representing visual decline in the long-term phase. Beta distributions were used for parameters representing probabilities and absolute utilities. Gamma distributions were used for parameters representing utility decrements, resource use and costs.

In addition, several scenario analyses were conducted. The univariate sensitivity analysis did not cover the transitions between health states occurring in the first year of the model [7, 8]. To account for uncertainty in treatment effectiveness, the assumption on the duration of the VN treatment effect was varied. In the base-case analysis the treatment effect was assumed to last for 40 years; in scenario analyses alternative durations of 7.5 years (which is the currently available Phase 1 trial follow-up data for VN), 20 years and life-long have been adopted. In another set of scenario analyses, alternative distributions have been used to model the visual decline of IRD patients with *RPE65* mutation during the long-term phase of the model. Also, alternative health state utilities (Table 2) were implemented in a scenario analysis; using Health Utilities Index Mark 3 (HUI3) estimates obtained from the same clinicians from which EQ-5D-5L estimates were obtained for the base-case [5]. In another scenario, health states were defined according to visual function only (in the base-case, health states were defined according to the worst of visual acuity or visual function). In further scenarios, alternative discount rates of 0% to 5% were applied. In another scenario analysis, we assigned health states according to the best-seeing eye, rather than the average eye. In an additional scenario analysis, for health state transitions with no data (from HS5), we assume patients remain in the same health state instead of assuming the movement is the same as the previous state.

We carried out two complementary analyses adopting a societal perspective. In the first, we included productivity losses of patients, by assuming patients of working age with severe visual impairment are 80% less productive than those without. This assumption was made based on a study from India [45]. In the second, we more conservatively assumed patients are 50% less productive as estimated from a Royal National Institute of Blind People (RNIB) study in the UK [23].

The original model that was adapted, was validated using standard procedures including tests to check model outputs were logical, and cell-by-cell checks of consistency and logic [23]. The model was implemented in Microsoft Excel 2010.

## Results

From the Swiss healthcare system perspective, the implementation of VN resulted in discounted strategy costs per patient of CHF 901’654 versus CHF 137’252 in the SoC strategy. The resulting incremental costs were CHF 764’402 per patient. Discounted QALYs per patient were 18.35 in the VN strategy and 11.62 in the SoC strategy, resulting in an increment of 6.73 QALYs per patient. This resulted in an ICER of CHF 113’526 per QALY gained for the VN strategy versus the SoC strategy (Table 5)

**Table 5:** Cost-effectiveness (CHF per patient) of VN versus SoC, discounted

**Effectiveness**	**SoC**	**VN**	**Difference**
Blindness-free years^a^	20.65	28.32	7.67
Quality-adjusted survival (QALYs)	11.62	18.35	6.73
**Costs**			
VN treatment	0	768’087	768’087
Eligibility testing (viable retinal cells)	0	493	493
Adverse events	0	768	768
Health care resource use^b^	137’252	132’305	-4’947
Total costs, healthcare system perspective	137’252	901’654	764’402
**Incremental cost effectiveness, health care system perspective (base case)**			
ICER (CHF per blindness-free year gained)^a^	99,603		
**ICER (CHF per QALY gained)**	**113’526**		

^a^ Counting health state HS5 as ‘blindness’.

^b^ Including costs of technical assistance, community care and residential care.

KEY: *CHF* Swiss francs, *ICER* incremental cost-effectiveness ratio, *QALYs* quality-adjusted life years, *SoC* standard of care, *VN* voretigene neparovec.

From the societal perspective, the VN and SoC strategies cost CHF 1’213’557 and CHF 977’006, respectively, yielding an increment of CHF 236’551 per patient. The ICER for the societal perspective was CHF 35’132 per QALY gained.

**Fig. 2:**
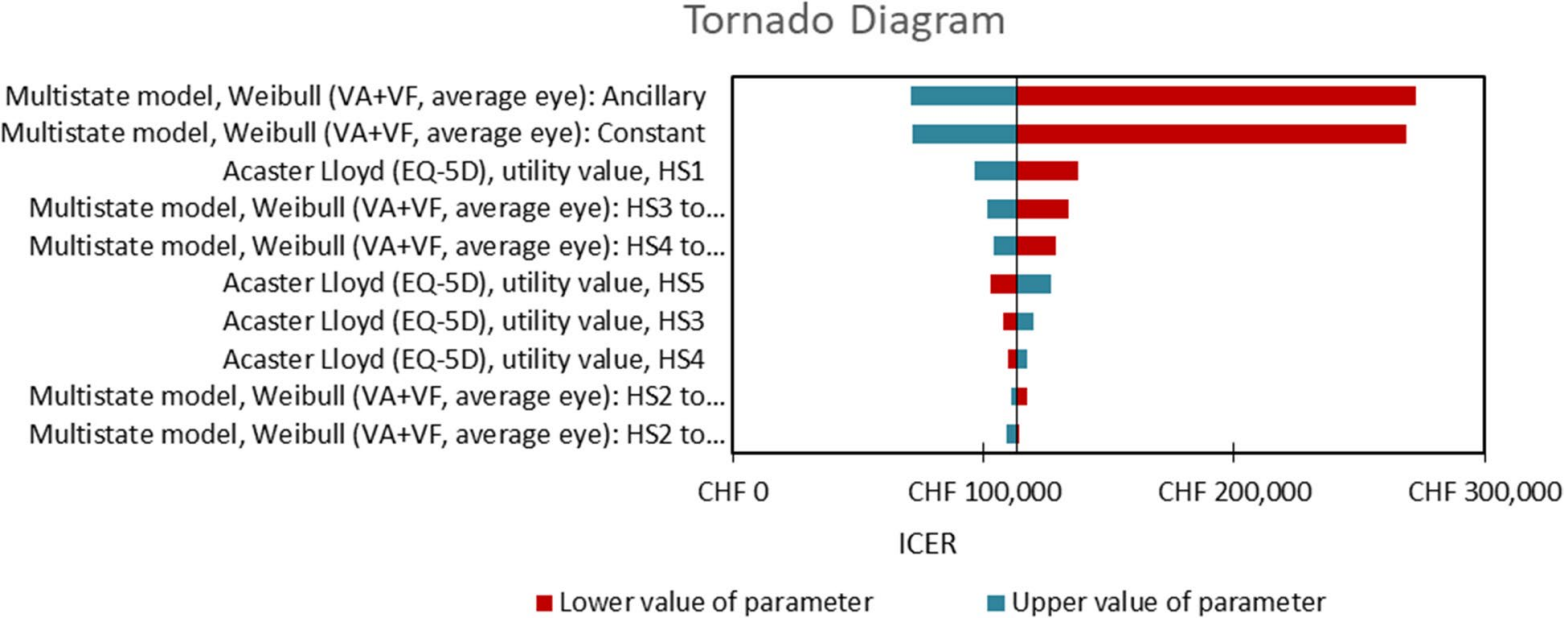
Tornado diagram for univariate sensitivity analysis. KEY: CHF, Swiss francs; HS, health state; ICER, incremental cost-effectiveness ratio; VA, visual acuity; VF, visual function

In the univariate sensitivity analysis for the healthcare system perspective, variation of the constant and ancillary parameters of the regression-based Weibull distribution reflecting the natural history model of long-term disease progression were the most influential (Fig. 2).

**Fig. 3:**
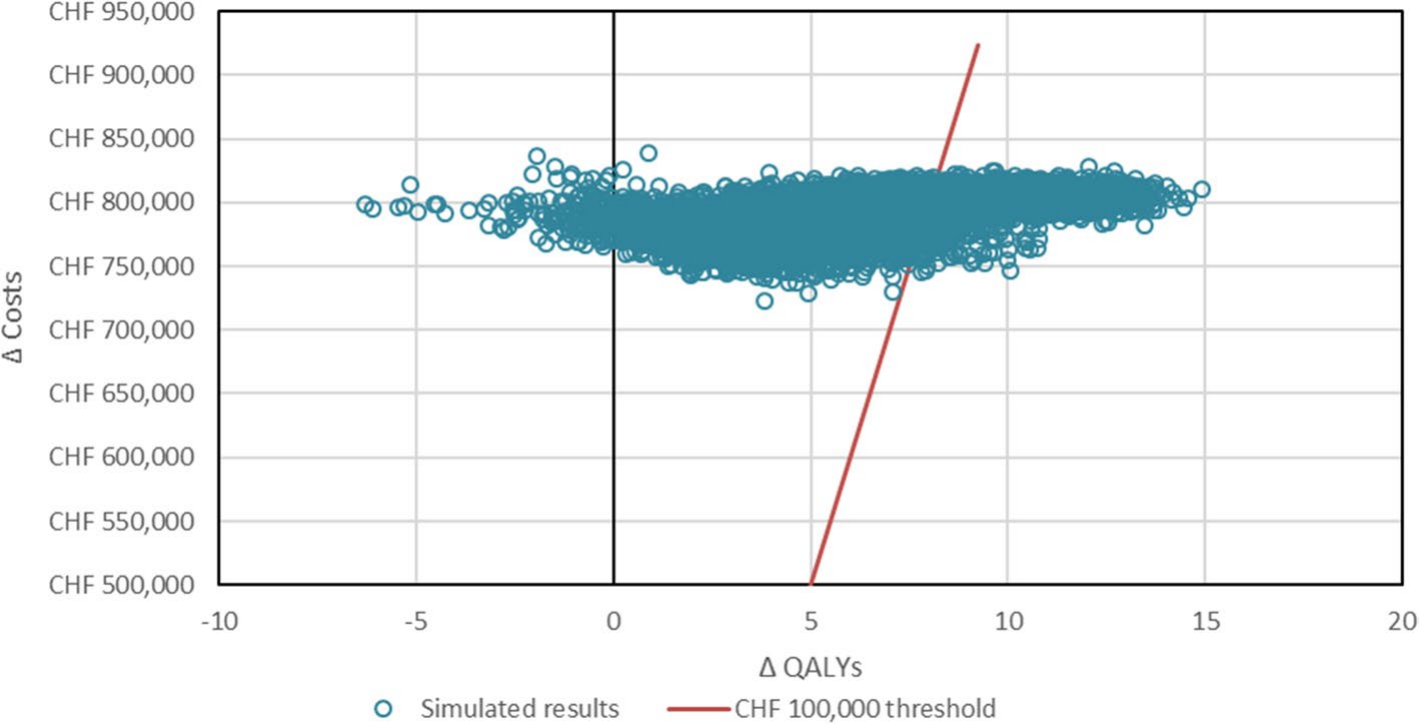
Incremental cost-effectiveness plane for the probabilistic sensitivity analysis. CHF, Swiss francs; QALYs, quality-adjusted life years; SoC, standard of care; VN, voretigene neparovec

**Fig. 4:**
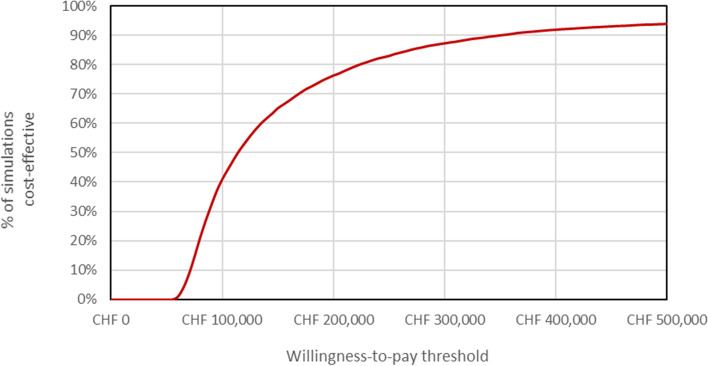
Cost-effectiveness acceptability curve for the probabilistic sensitivity analysis. CHF, Swiss francs; CEAC, cost-effectiveness acceptability curve; QALYs, quality-adjusted life years; SoC, standard of care; VN, voretigene neparvovec

At an assumed cost-effectiveness threshold of CHF 100,000 per QALY gained, the PSA-based probability of being cost-effective was 41%, for the healthcare system perspective (Figs. 3 and 4).

Results from analyses from a societal perspective and scenario analyses are presented in Table 6. The analyses from a societal perspective resulted in ICERs of CHF 35’132 per QALY gained when assuming an 80% productivity loss, and CHF 62’947 per QALY gained when assuming a 50% productivity loss. Assuming a duration of the treatment effect of VN of 7.5 years, 20 years or life-long, compared to 40 years in the base case, resulted in ICERs of CHF 275`213, CHF 156'171 and CHF 96'384 per QALY gained, respectively. Applying a discount rate of 0% generated an ICER of CHF 39'767 per QALY gained, and a discount rate of 5% generated an ICER of CHF 193'548 per QALY gained.

**Table 6:** Results from a societal perspective and of scenario analyses

Description of scenario	VN cost (CHF)	SoC cost (CHF)	Incremental cost of VN (CHF)	VN QALYs	SoC QALYs	Incremental QALYs of VN	ICER (CHF/QALY)
Base-case analysis	901’654	137’252	764’402	18.35	11.62	6.73	113’526
Societal perspective; 80% productivity loss	1,213,557	977,006	236,551	18.35	11.62	6.73	35,132
Societal perspective; 50% productivity loss	1,118,565	694,728	423,837	18.35	11.62	6.73	62,947
VN treatment effect lasts for 7.5 years	907,077	137’252	769’825	14.42	11.62	2.80	275,213
VN treatment effect lasts for 20 years	907,427	137,252	770,175	16.55	11.62	4.93	156,171
VN treatment effect lasts for lifetime	853,424	137,252	716,173	19.05	11.62	7.43	96,384
Health state assignment based on VF only	900,423	137,325	763,097	18.86	14.01	4.86	157,157
Extrapolation for long-term phase: Gompertz	900,394	137,264	763,130	18.28	10.96	7.32	104,198
Extrapolation for long-term phase: Log-logistic	901,457	137,230	764,227	18.38	12.24	6.15	124,339
Extrapolation for long-term phase: Log-normal	901,460	137,229	764,230	18.36	12.40	5.95	128,341
Extrapolation for long-term phase: exponential	894,184	137,038	757,146	18.54	13.28	5.26	143,885
Health state utilities obtained using HUI3 [5]	901,654	137,252	764,402	12.19	4.87	7.32	104,413
Discount rate 0%	1,343,226	600,567	742,659	40.42	21.74	18.68	39’767
Discount rate 5%	833,304	65,380	767,924	12.64	8.67	3.97	193,548
Best seeing eye used to assign health states	900,262	137,282	762,980	18.51	11.47	7.04	108,378
Where no transition observed, assume patients remain in same health state.	901,654	137,252	764,402	18.10	11.52	6.58	116,177

KEY: *CHF* Swiss franc, *HUI3* Health utilities index mark 3, *ICER* incremental cost-effectiveness ratio, *QALY* quality-adjusted life year, *SoC* standard-of-care, *VF* visual function, *VN* voretigene neparvovec

In the base case, health state membership was assigned based on the worst of VA/VF in the patient, using the average of both eyes. When health state membership was alternatively assigned using VF only, the ICER increased to CHF 157'157 per QALY gained. Using alternative distributional assumptions for modelling visual decline in the long-term did not change the base case ICER substantially. Use of alternative health state utility values estimated using the HUI3 instead of the EQ-5D-5L resulted in an ICER of CHF 104,413 per QALY gained.

## Discussion

The cost-effectiveness analysis of VN, a novel treatment for IRD patients with biallelic *RPE65*-mutation, resulted in an ICER of CHF 113’526 per QALY gained from the healthcare system perspective. Calculating costs from a societal perspective changed the cost-effectiveness substantially, mainly due to the avoidance of loss of patient productivity during adulthood. When assuming that the productivity of a person with at least severe visual impairment is 20% or 50% of that of a person without or with moderate visual impairment, the resulting ICERs were CHF 35’132 and 62’947 per QALY gained respectively. There are approximately 13 patients in Switzerland currently eligible for VN treatment, and the net undiscounted incremental cost to the Swiss health care system of treating these 13 patients over their lifetime is estimated to be CHF 9,654,567 (mean undiscounted incremental costs from this study multiplied by 13 patients).

In terms of cost-effectiveness thresholds adopted in Switzerland, for some interventions a threshold of CHF 100,000 per QALY, from the perspective of the statutory health insurance, has been tentatively assumed in previous analyses [47–49]. However, for ultra-orphan diseases, it may be appropriate to consider a higher threshold; for instance NICE in the United Kingdom recognises thresholds ranging from GBP 100,000 to 300,000 (CHF 122,000 to 366,000) depending on the size of treatment effect, for ultra-orphan diseases [50]. These thresholds are much higher than the GBP 20,000 to 30,000 threshold range that NICE typically adopts for diseases which are not very rare.

These results were affected by a substantial degree of uncertainty. This is unavoidable given small patient numbers, the rareness of the disease, and still limited observation periods. In terms of costs, the treatment costs of VN and, from the societal perspective, the avoided loss of patient productivity, were the dominant drivers of the difference between the VN and SoC strategies. The impact of all other cost elements was relatively marginal. In terms of effectiveness, the key assumption underlying the base case ICER result from the healthcare system perspective, and also of the ICER from the societal perspective, was the assumption of a duration of VN treatment effect of 40 years. The uncertainty around the long-term effect of the treatment is a limitation of this analysis. If the duration of the treatment effect was substantially shorter (20 years), the ICER would increase substantially (e.g. to CHF 156,171 per QALY gained from the healthcare system perspective). On the other hand, if the durability of the treatment is life-long, the ICER would decrease substantially. In a phase 1 trial of VN in 12 patients who were followed up for 7.5 years after VN treatment, improvements in visual function were observed to be sustained throughout the period [24]. Another limitation of this study is that due to the wide scope of costs generated from visual impairment not all have been captured; for instance the cost of treating depression resulting from blindness or visual impairment. In the scenario analyses for the healthcare perspective, influential parameters included the alternative assumptions on utility estimates and choice of distributional assumption for the estimation of transition probabilities representing natural history of disease in the Markov part of the cost-effectiveness model. The Weibull distribution assumed in the base case generated more favourable results than other assumptions, but was also statistically most plausible [25].

A further limitation was that age-dependent utility was not accounted for. Reliance on international parameters for estimation of productivity losses in patients of working age with severe visual impairment [23, 45], was another limitation of this analysis, but was necessary due to an absence of Swiss sources, and that no better sources were identified. It may be considered that the UK study provides a better approximation of the productivity losses in Switzerland compared with the Indian study, as the UK study is more recent and the UK has a more similar economic and social structure to Switzerland. Working age was assumed to begin at age 18 in the UK study, and at age 15 in the Indian study. We would propose undertaking a future study in Switzerland (e.g. survey-based) to provide more recent and relevant data for Switzerland on productivity losses in patients resulting from blindness/visual impairment.

Wide variation in the size of estimated VN treatment effect was observed in cost-effectiveness analyses published in other countries. These estimates range from 2.7 discounted incremental QALYs in a US Institute for Clinical and Economic Review report (leading to an ICER of 287’915 United States Dollars per QALY gained) [6], 4.0 discounted incremental QALYs in the preferred analysis of the evidence review group for the NICE VN assessment for the UK (leading to an ICER of Great British Pounds 155’750 per QALY gained) [23], 6.4 discounted incremental QALYs in the UK economic evaluation based on the same model as the present analysis (leading to an ICER of Great British Pounds 95’072 per QALY gained) [22], to 9.5 discounted incremental QALYs in an US economic evaluation published by Johnson et al (leading to an ICER of 79’618 United States Dollars per QALY gained from a health care perspective) [51]. The estimated discounted incremental QALYs in analyses performed for Canada [52], Denmark [53], Germany [54], and the Netherlands [55], also fall within this range.

There were several methodological differences between these studies, including the discount rates used and the assumed duration of VN treatment effect [56]. Importantly, compared with this study which assumed a duration of VN treatment effect of 40 years, in the Institute for Clinical and Economic Review report, a maximum duration of treatment effect of 20 years was assumed [6] and a lifelong duration of treatment effect was assumed by Johnson et al [51]. A 40 year duration of VN treatment effect was assumed by the evidence review group for the NICE VN assessment.

## Conclusion

Assuming a public price of VN of CHF 759’968, the incremental cost-effectiveness ratio of the VN strategy compared to the SoC strategy is estimated to be CHF 113’526 per QALY gained from a Swiss healthcare system perspective. Furthermore, ICERs of CHF 35'132 and 62'947 per QALY gained were estimated from the societal perspective assuming a 80% and 50% productivity loss from visual impairment in working-age patients, respectively. There is substantial uncertainty present in these estimates, most importantly driven by the clinical assumptions related to the lasting effects of treatment. This analysis aims to provide information to Swiss decision makers on whether or not VN is cost-effective at a public price of CHF 759'968.

## Supplementary Information

**Additional file 1:** **Appendix table 1.** Description of VA/VF-based health states. **Appendix table 2.** Multistate model parameters based on different distributions, used to calculate transition probabilities between health states (HSs) for the long-term phase.

## Data Availability

The datasets generated and/or analysed during the current study are not publicly available due to copyright, but are available from the corresponding author on reasonable request. Acceptance of the request is subject to approval from Novartis Pharma AG.
