# Supplementary material for: Cost-effectiveness of voretigene neparvovec in the treatment of patients with inherited retinal disease with RPE65 mutation in Switzerland
Source: BMC Health Serv Res. 2022 Jun 28;22:837. doi: 10.1186/s12913-022-08211-y (PMC9241179; doi:10.1186/s12913-022-08211-y)
Supplement: Supplementary file 1 — Additional file 1: Appendix table 1. Description of VA/VF-based health states. Appendix table 2. Multistate model parameters based on different distributions, used to calculate transition probabilities between health states (HSs) for the long-term phase. [file 12913_2022_8211_MOESM1_ESM.docx]

Appendix table 1. Description of VA/VF-based health states

|  | **Based on the average score of both eyes, the worst of:** | | |
| --- | --- | --- | --- |
|  | **Visual Acuity (VA) (LogMAR)** |  | **Visual Field (VF) (sum total degrees)** |
| HS1: Moderate VI | VA<1 | Or | VF>240 |
| HS2: Severe VI | VA≥ 1 and VA<1.4 | Or | VF≤ 240 and VF>144 |
| HS3: Profound VI | VA≥ 1.4 and VA<1.8 | Or | VF≤ 144 and VF>48 |
| HS4: Count Fingers | VA≥ 1.8 and VA<3 | Or | VF≤ 48 |
| HS5: Hand Motion to No Light Perception | VA≥ 3, or indications of HM, LP, NLP across both eyes |  | - |
| Death | - |  | - |

HM, hand motion; HS, health state; LP, light perception; NLP, no light perception; VA, visual acuity; VF, visual field; VI, visual impairment.

Appendix table 2. Multistate model parameters based on different distributions, used to calculate transition probabilities between health states (HSs) for the long-term phase

| Parameter | Weibull (base-case) | Gompertz | Log-logistic | Log-normal | Exponential |
| --- | --- | --- | --- | --- | --- |
| HS1 to HS3 | -2.48 | -2.48 | 1.41 | 1.42 | -2.48 |
| HS1 to HS4 | -2.48 | -2.48 | 1.39 | 1.09 | -2.48 |
| HS1 to HS5 | -18.30 | -17.25 | 8.48 | 5.38 | -17.31 |
| HS2 to HS3 | -0.60 | -0.51 | 0.51 | 0.53 | -0.34 |
| HS2 to HS4 | -3.00 | -2.91 | 1.69 | 1.64 | -2.74 |
| HS2 to HS5 | -18.32 | -17.28 | 8.56 | 5.44 | -17.31 |
| HS3 to HS4 | -1.37 | -1.40 | 0.94 | 0.96 | -0.80 |
| HS3 to HS5 | -18.36 | -17.37 | 8.77 | 5.75 | -17.31 |
| HS4 to HS5 | -1.55 | -1.56 | 1.00 | 1.08 | -1.01 |
| Constant | -14.95 | -9.21 | 7.99 | 8.02 | -8.59 |
| Ancillary | 0.59 | 0.00 | -0.79 | -0.14 | - |
| *AIC (model diagnostic)* | 166.3636 | 169.6609 | 166.6239 | 169.9392 | 180.9312 |
| *BIC (model diagnostic)* | 206.4635 | 209.7608 | 206.7238 | 210.0391 | 217.3856 |

HS, health state. *Hazard ratios can be obtained by exponentiating the coefficients for HS1 to HS3, HS1 to HS4 etc.. The AIC (Akaike information criterion) and BIC (Bayesian information criterion) are for the full statistical model.
